# Assessment of soil metal exposure, associated health risks and indoor dust screening in early learning programmes in Gauteng Province, South Africa

**DOI:** 10.1007/s10653-026-03034-w

**Published:** 2026-02-04

**Authors:** Tahira Kootbodien, Yonela Mkunyana, Melissa Nel, Nomfundo Mahlangeni, Renee Street

**Affiliations:** 1 Environment and Health Research Unit, South African Medical Research Council, Francie Van Zyl, Tygerberg, Cape Town, South Africa; 2 Centre for Environmental and Occupational Health Research, School of Public Health and Family Medicine, University of Cape Town, Observatory, Cape Town, 7925 Western Cape South Africa; 3 Environmental Health, University of Johannesburg, Doornfontein Campus, Johannesburg, South Africa

**Keywords:** Early learning programme, Metal exposure, Soil, Dust, IEUBK model

## Abstract

Exposure to heavy metals is a global health concern, especially for children under the age of five. In South Africa, industrial and mining activities have contributed to environmental accumulation of metals. Early learning programmes (ELPs) or preschools are primary spaces for learning and play, making them critical for mitigating early life metal exposure. This study examined arsenic (As), cadmium (Cd) and lead (Pb) levels in soil and dust from selected ELPs in two metropolitan municipalities in Gauteng Province, South Africa, to assess potential exposure risks for children under five. As part of a nationally represented survey of early childhood outcomes, 70 ELPs were stratified into three fee bands (low, medium, high). Soil and dust samples were collected from outdoor play areas and indoor classrooms and analysed using inductively coupled plasma-optical emission spectroscopy (ICP-OES) and inductively coupled plasma-mass spectrometry (ICP-MS). Dust results were semi-quantitative (no surface area measured) and not used in risk calculations. Potential Pb exposure in children was evaluated using the US EPA Integrated Exposure Uptake Biokinetic (IEUBK) model to predict blood Pb levels. Geographic Information System (GIS) mapping identified spatial patterns and hotspots of metal concentrations relative to potential pollution sources. Soil Pb levels were below South African reference values; however, 9.0% of samples exceeded Canadian guidelines, while dust Pb was detected in all samples. Soil As was detected in 95% of samples, with higher concentrations in low-fee schools (*p* = 0.002); 10.7% exceeded Canadian guidelines. Cd concentrations were low across all sites. Estimated As exposures suggested minimal non-carcinogenic health risk to children through soil ingestion, while IEUBK modeling predicted a geometric mean blood Pb levels of 1.72 µg/dL (95% CI 0.69–4.31), with 6.6% exceeding the CDC blood lead reference value. Children exposed to As at school for approximately 2 years had a combined lifetime cancer risk of ~ 2.2 × 10⁻^4^, exceeding the USEPA’s acceptable threshold. Hotspot and kernel density estimation analysis identified localised elevated soil As and Pb levels near areas of concentrated mining activity, indicating legacy industrial sources as likely contributors. Localised hotspots of Pb and As highlight the need for continued environmental monitoring and targeted interventions to ensure safe learning environments for young children, particularly given the carcinogenic risk associated with As exposure.

**Supplementary Information:**

The online version contains supplementary material available at 10.1007/s10653-026-03034-w.

## Introduction

Children under the age of five are highly susceptible to exposure and adverse effects from toxic metals, such as arsenic (As), cadmium (Cd) and lead (Pb) (Bellinger, 2004; Landrigan et al., 2019). Children in this age group are at a crucial stage of development, where their bodies and brains are rapidly growing and forming. Children in low- and middle-income countries (LMICs) are disproportionately affected, as they are already vulnerable due to poor nutrition, inadequate hygiene and sanitation, unsafe water, and limited access to healthcare (Landrigan et al., 2019; World Health Organization). Compared to adults, children are more susceptible to toxic metal exposure due to hand-to-mouth behaviour, immature metabolic pathways, and the vulnerability of early developmental processes, which are easily disrupted (Bellinger et al., 1991; Landrigan & Goldman, 2011).

These existing vulnerabilities are magnified when industrial activities and other pollution sources are situated near where children live and attend school, intensifying their overall health risks. In South Africa, poor town planning practices, industrial growth and urbanisation have led to the location of various industrial processes such as mining operations, battery manufacturing and recycling and smelting operations within or in close proximity to residential areas (Sanders et al., 2014). The emission of pollutants such as heavy metals into air, water and soil may cause significant local and downstream contamination, as well as harmful exposures in affected communities (Harper et al., 2003). The World Health Organization estimates that nearly half of the 2 million lives lost to known chemicals exposure in 2019 were due to exposure to lead (World Health Organization, 2021). Pb exposure is estimated to account for 21.7 million years lost to disability and death (disability-adjusted life years, or DALYs) worldwide due to long-term effects on health, including 30% of the global burden of idiopathic intellectual disability, 4.6% of the global burden of cardiovascular disease and 3% of the global burden of chronic kidney diseases (World Health Organization, 2021). Widespread environmental Pb exposure can contribute to adverse neurodevelopmental outcomes in children, which is associated with substantial economic losses (Landrigan et al., 2002). The detrimental neurodevelopmental effects of both prenatal and childhood Pb exposure include decreased intelligence quotient (IQ) and cognitive function, diminished attention span, academic proficiency, fine-motor control, and visual-motor control (Bellinger et al., 1991). A recent review by Kinally and colleagues (2025) which analysed 39 studies from 26 countries, primarily low- and middle-income, quantified contributions of specific lead sources to blood Pb levels (Kinally et al., 2025). Findings showed that the largest average blood Pb levels were associated with industrial pollution hotspots (42.3 μg/L), occupational exposures (31.4 μg/L), and deteriorated paint (28.0 μg/L), with additional contributions from traditional medicines and cosmetics, food ware, smoking, foods, and geophagy (Kinally et al., 2025). Consistent with evidence that no safe level of lead exposure exists, the CDC revised the childhood blood Pb reference value to 3.5 µg/dL in 2021 (Ruckart et al., 2021). More recently, the USEPA introduced stricter dust-related clearance levels for Pb (US EPA, 2025a), reflecting ongoing efforts to reduce childhood Pb exposure.

Pb exposure often coincides with other toxic metal exposures, such as As and Cd, due to co-contamination of air, soil, or water supplies by industrial activities (Diazbarriga et al., 1993). Co-exposure to toxic metals is associated with adverse health outcomes including birth defects (Diazbarriga et al., 1993), decreased neuropsychological function, IQ, and overall academic achievement (Bellinger et al., 1991; Wright et al., 2006). Exposure to a mixture of metals has been shown to have greater toxicological effects in animal studies than exposure to a single metal (Pecze et al., 2005). However, there have been limited studies of the interactive effects of toxic metals resulting from co-exposure in children living in LMICs.

Efforts in early childhood development in recent years meant that more children have gained access to child-care and educational programmes, especially through community-based and home-based centres (Atmore, 2013; Department of Basic Education, 2022). However, in low-income areas situated near industrial facilities and cottage industries, affected communities are exposed to harmful metals through the air and soil, causing adverse health and social impacts (Bose-O’Reilly & Landrigan, 2022). While studies on heavy metal exposure in South Africa are available (Mathee et al., 2018, 2022), evidence specific to early learning programmes (ELPs), particularly soil and dust measurements and associated health risk estimates, remain limited (Cindi et al., 2020). To fill this gap, this study aimed to assess the metal exposure (As, Cd and Pb levels) in soil and dust and associated health risks at selected ELPs in Gauteng Province, South Africa and evaluate associated health risks via children’s ingestion of soil and dust. The study also sought to identify spatial patterns of contamination in relation to pollution sources across sites. A risk assessment framework was applied, using non-carcinogenic hazard quotients and lifetime cancer risk for As and US EPA IEUBK modeling to estimate predicted blood Pb levels, with dust measurements primarily used for screening and site comparison.

## Methods

### Study setting and data sources

**Fig. 1:**
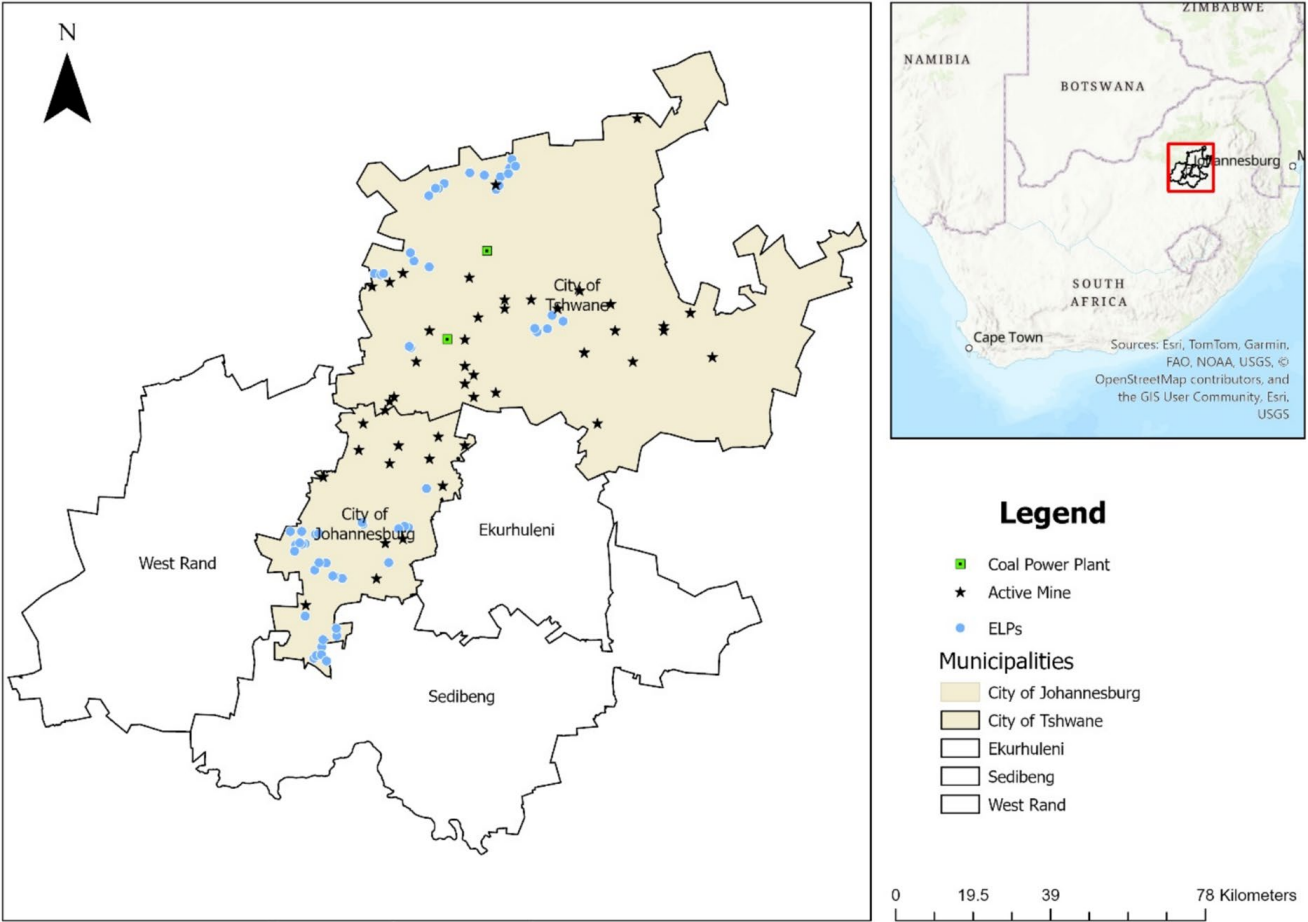
Map of the study area (Gauteng Province, South Africa), showing location of ELPs (blue circle) and proximity to select industrial sources of pollution (coal-fired power plants and active mines)

This environmental assessment study was conducted in November 2024 in Gauteng Province, South Africa, across a sample of ELPs from two metropolitan municipalities (City of Tshwane and City of Johannesburg). From a total of 275 ELPs in Gauteng Province, participating in a nationally representative survey of 1388 ELPs (Giese et al., 2022), a subsample of 70 ELPs was randomly selected, and further stratified by fee band based on annual tuition fees to account for socioeconomic diversity, classified as: low: < ZAR 350 per month, middle: ZAR 350–550 and high: > ZAR 550 (1 ZAR = 0.057 USD). These fee bands are used as proxies for the probable socio-economic background of the children enrolled in ELPs. The selected ELPs were in areas with varying degrees of industrial activity. Figure 1 shows the spatial distribution of study sites in relation to major pollution sources, including active mines and coal-fired power plants, which may contribute to localised environmental contamination and potential exposure risks for young children. Data on selected pollution sources, including locations of active mines were sourced from the Department of Mineral Resources and Energy (Department of Mineral Resources & Energy, 2024), coal-fired power plants from Global Coal Plant Tracker Dashboard (Global Energy Monitor, 2024).

#### Environmental sampling and data collection

*Outdoor soil collection:* Two soil samples were collected at each school (ELP sites) following the United States Environmental Protection Agency (US EPA) guidelines (US EPA, 2012). Sampling locations included common play areas such as outdoor playgrounds, sandpits, where children spend most of their time, and in the absence of soil on the school grounds, nearby pavements. About 1 g of air-dried and ground sample was digested in aqua regia (a 3:1 (v/v) mixture of 35% hydrochloric acid (HCl) and 69% nitric acid (HNO_3_)) under reflux conditions at 112.5 °C, following US EPA Method 3050B. This digestion method represents a strong acid extraction and does not dissolve silicate-bound fractions; therefore, reported concentrations reflect pseudo-total, environmentally available metal content rather than total soil concentrations. Analysis of As, Cd and Pb was conducted using Inductively Coupled Plasma-Optical Emission Spectrometry (ICP-OES, iCAP™ 6000 Series, Thermo Fischer, USA) according to the US EPA Method 6010D, with emission wavelengths of 189.042 nm (As), 228.802 nm (Cd) and 220.352 nm (Pb). Calibration was performed using GB19 Multi-Element Calibration Standard PrimAg Plus (ROMIL, Batch JE742403), along with 10,000 ppm single-element standards of Fe and Al (PrimAg Plus, ROMIL). Quality assurance and quality control procedures included analysis of procedural blanks, duplicate samples, matrix spike recoveries, and periodic calibration verification checks; blank concentrations were below detection limits and spike recoveries were within acceptable analytical ranges. Method validation included assessment of limit of detection, reproducibility and the linear dynamic range. While no soil-based certified reference material was analysed, quantification was supported by traceable calibration standards and routine QA/QC checks. Arsenic was quantified by ICP-OES, and potential spectral or matrix-related interferences at low concentrations are acknowledged as a study limitation.

*Indoor surface dust collection*: Two surface dust wipe samples were collected from indoor areas at each ELP site, using ghost wipes. Sampling targeted common contact surfaces in indoor play areas, including floors, learning/play tables, and windowsills. As no defined surface area was measured, the results are considered semi-quantitative and were used for screening purposes to identify potential surface contamination. The digestion of the wipes was conducted in accordance with the Occupational Safety and Health Administration method ID-125G (OSHA, 2002). Briefly, dust wipes were placed in a beaker and left to stand in 4 mL of 1:1 sulfuric acid (H_2_SO_4_) solution for 5 min. This was followed by the addition of 2 mL of deionized water and 2 mL of concentrated HNO_3_, and the mixture was allowed to stand for 15 min. An additional 8 mL of HNO_3_ was then added, and the sample was digested on a hot plate until the solution turned brown. Hydrogen peroxide (H_2_O_2_) was added dropwise until the solution became clear. After cooling, 4 mL of HCl was added to enhance the dissolution of the elements. The solution was reheated to near boiling, cooled to room temperature, and transferred to a 50 mL volumetric flask, which was then made up to volume with deionized water. Element analysis was performed using Inductively Coupled Plasma–Mass Spectrometry (ICP-MS) according to US EPA Method 6020B. Calibration standards were prepared from a 1000 mg/L, multi-element calibration stock solution. Quality control procedures included the use of reagent blanks, assessment of detection limits and calibration linearity (R^2^ ≥ 0.995). The accuracy and reproducibility of the analytical procedure were assessed using blank ghost wipes and the Standard Reference Material (SRM) from the National Institute of Standards and Technology (NIST SRM 2583—Trace Elements in Indoor Dust). Each SRM sample was digested and analysed in triplicate (n = 3) under the same conditions as the field samples to assess reproducibility. The measured values for Cd, Cr and Pb in the SRM were in good agreement with the certified values (Table S1).

*Facility-based questionnaire:* A structured facility-based questionnaire was administered to the principal of each ELP that captured environmental and infrastructural characteristics of each site. Information collected included building type, availability of outdoor play areas, meal preparation practices, types of fuel used for cooking and main source of power. Information was also collected on washing and sanitation facilities, i.e. main water source, handwashing infrastructure and toilet facilities available for children.

#### Exposure assessment

We conducted a human health risk assessment following the US EPA guidelines (USEPA, 1989; 2002), adapted for the South African early childhood context for As and applied the US EPA Integrated Exposure Uptake Biokinetic (IEUBK) model to predict blood lead concentrations in children aged 4–5 years (US EPA, 2021). Cd was not quantitively assessed because concentrations were below the limit of detection (LOD) in 83.6% of samples. Due to the high censoring, hazard quotient calculations for Cd were not performed. Instead, Cd results were evaluated qualitatively based on detection frequency.

The assessment focused on children aged 4–5 years, who are more vulnerable to environmental exposures due to higher soil and dust ingestion rates and lower body weights.

*Average Daily intake (ADI):* For As, the average daily intake (ADI) through soil ingestion was estimated using the following equation:

$$ADI = \frac{Metal concentration x Ingestion Rate x Exposure Frequency x Exposure Duration}{Body Weight \left(kg\right) x Average time (days)}$$

where:

metal soil concentration (mg/kg), ingestion rate is the ingestion rate of the child per day (100 mg.day^−1^ for children aged 4–5 years), exposure frequency (180 days/per year, assuming school weekday attendance), exposure duration (2 years), body weight (average body weight of a child aged 4–5 years, 16 kg) and averaging time (period over which the dose is averaged for non-cancer outcome: 730 days).

#### Risk calculations

The hazard quotient (HQ) was used to calculate the non-carcinogenic risk, and the calculation equation recommended by the US EPA was as follows:

$$Hazard Quoteints \left(HQ\right)=\frac{ADI}{Reference dose for each metal (mg/kg)}$$

where reference dose is maximum tolerable daily intake of a specific metal (mg/kg). The reference dose for As and is 0.0003.

$$Hazard Index \left(HI \right)=\sum HQ$$

A hazard index (HI) was derived by summing HQs for each site. An HI > 1 indicates potential for non-carcinogenic adverse health effects.

We estimated the lifetime cancer extra risk for children exposed to As for approximately up to 3 years, while attending ELPs, using the linear cancer slope factors (US EPA, 2025b). Calculations accouted for partial lifetime exposure (3 years of a 70 year lifetime) and an age dependent adjustment factor (moderate sensitivy, 2–16 years) for early life exposure to carcinogens. We used the linear cancer slope factor model, as the daily estimated intake calculated for As (0.0814 μg/kg-day) was below the 0.2 μg/kg-day threshold.

The IEUBK model was used to estimate predicted blood Pb levels in children, integrating multiple exposure pathways including soil and indoor dust ingestion, dietary intake, drinking water, and air (US EPA, 2021). The model predicts the geometric mean blood Pb level and the probability of a child exceeding the CDC blood lead reference value of 3.5 µg/dL (Ruckart et al., 2021). The IEUBK model runs were executed in batch file processing feature of v2.0 (build 1.72), with each row representing potential children aged 4–5 attending the ELP (US EPA, 2021). Predicted blood Pb levels were summaried as geometric mean and 95% CI and stratified by fee band and municipality. The proportion of predicted values exceeding the CDC blood Pb reference level of 3.5 µg/dL was estimated.

### Spatial analysis

#### Kernel density estimation (KDE)

To assess the spatial distribution of potential pollution sources, a KDE analysis was applied using ArcGIS (Pro Standard—Online, ESRI, Redlands, CA, USA). The KDE shows intensity of point features per unit area by fitting a smoothly tapered surface over each observation point. In this study, mines and coal-fired power plants were geocoded and weighted (mines = 1 and coal power plants = 2) and used as input point features. A search radius of 1 km (determined using average nearest neighbour) was applied, reflecting a distance over which pollution sources could realistically influence children’s exposure at the schools. Alternative radii (e.g., 0.5 km and 2 km) were evaluated and produced similar spatial patterns, indicating that results were robust to reasonable parameter variations. Density values were calculated as pollution points per square kilometer. The resulting raster surfaces were classified into low, medium, and high-density zones, highlighting areas with concentrated pollution sources.

### Hotspot analysis

To identify statistically significant spatial clusters of elevated metal concentrations, hotspot analysis was performed using the Getis-Ord Gi* statistic in ArcGIS. This method evaluates each sampling location in the context of its neighbours, determining whether high or low values cluster spatially more than would be expected by random chance. Soil concentrations of As and Pb were analysed separately. The analysis was conducted at a fixed distance band of 1 km (based on average nearest-neighbour distance), and statistical significance was assessed at 90%, 95%, and 99% confidence levels. Hotspots (areas with significantly higher concentrations) and cold spots (areas with significantly lower concentrations) were mapped to visualise spatial clustering patterns. Together, KDE and hotspot analyses enabled the identification of regions where elevated soil metal concentrations co-occur with high densities of pollution sources.

### Statistical analysis

Descriptive statistics and risk calculations were performed using Stata version 18.0 (StataCorp LLC, College Station, TX, USA). Data were summarised using detailed, descriptive statistics. Continuous variables were expressed as mean ± standard deviation (SD) or as median with an interquartile range (IQR), and categorical variables are reported as count and percentages. Schools were analysed according to the three fee bands (low, medium and high) based on their annual tuition fees, serving as a proxy for socioeconomic status (SES). Municipality was included as a stratifying variable in the analysis to examine spatial trends in metal concentrations and associated risks.

Limits of detection (LOD) for soil analysis were 5 mg/kg for Pb, 0.5 mg/kg for As, and 0.1 mg/kg for Cd. The LOD for Pb was 5.0 mg/kg, with 8.2% of samples below this limit. For As, the LOD was 0.5 mg/kg, with 4.9% of samples below the limit. Values below the LOD were replaced with half the LOD (2.5 mg/kg for Pb and 0.25 mg/kg for As). For Cd, where 83.6% of samples were below the LOD, concentrations were analysed as a binary variable (detected vs. not detected) to reduce bias associated with substitution methods. Dust metal concentrations were reported as detection frequencies (%) because samples were collected from elevated surfaces such as bookshelves and windowsills rather than floors, which were frequently cleaned. Comparisons between groups of metal concentrations and health risk metrics were conducted by school fee band (low, middle and high) and municipality. Differences in continuous outcomes across fee bands and municipalities were assessed using analysis of covariance (ANOVA) or Kruskal–Wallis test, as appropriate. Comparisons of percentages were performed using chi-square test. Metal concentrations were summarised as means with 95% confidence intervals. Metal concentrations were also explored in relation to the ELPs’ socioeconomic status (fee band) and geographic location.

### Ethical approval

Permission to conduct the national Thrive by Five Index 2024 study was obtained from the National Department of Basic Education. Ethical approval was obtained from the Ethics Review Committee of the Faculty of Humanities, University of Cape Town (PSY2024-032), and the Human Research Ethics Committee (HREC) of the South African Medical Research Council (SAMRC).

## Results

Table 1 summarises the characteristics of the 70 participating ELPs, stratifying by fee band and municipality, as well as concentrations of As, Cd and Pb in soil samples and associated health risk indicators. Although fewer ELPs were in the high fee band category, this difference was not statistically significant across municipalities (*p* = 0.373). Most schools were conventional brick buildings (87%), used electricity as the main power source (90%), and had tap water within the building (87%), with minimal variation across fee categories. Informal housing and use of alternative power (solar or paraffin) were more common in low-fee schools. Handwashing facilities varied; while taps were most common (57%), low-fee schools relied more on bowls/buckets, whereas high-fee schools had greater access to taps. Most schools (87%) had outdoor play areas, with no difference by fee band. No significant differences were observed across fee categories for any of the characteristics.

**Table 1:** Description of early learning programmes (ELP) characteristics, environmental metal concentrations, and health risk indicators stratified by school fee band in Gauteng, South Africa

Characteristic	All	Low fee band	Middle fee band	High fee band	p-value
*Number of schools*	70	28 (40.0)	25 (35.7)	17 (24.3)	
*Municipality*					
Johannesburg	35 (50.0)	13 (37.2)	11 (31.4)	11 (31.4)	
Tshwane	35 (50.0)	15 (42.9)	14 (40.0)	6 (17.1)	0.373
*N (soil samples)*	122	53	45	24	
*N (dust samples)*	136	55	47	34	
*Building type*					
Conventional brick building	59 (86.8)	23 (82.1)	21 (91.3)	15 (88.2)	0.372
Prefab building	4 (5.9)	1 (3.6)	2 (8.7)	1 (5.9)	
Informal housing	5 (7.3)	4 (14.3)	0 (0)	1 (5.9)	
*Main source of power*					
Electricity	61 (89.7)	23 (82.1)	21 (91.3)	17 (100)	0.249
Gas	3 (4.4)	1 (3.6)	2 (8.7)	0 (0)	
Solar	2 (2.9)	2 (7.1)	0 (0)	0 (0)	
Paraffin	2 (2.9)	2 (7.1)	0 (0)	0 (0)	
*Main source of water*					
Tap water in the building	59 (86.8)	23 (82.1)	21 (91.3)	15 (88.2)	0.548
Tap water onsite/outside	5 (7.4)	1 (3.6)	2 (8.7)	2 (11.8)	
Public/communal tap	2 (2.9)	2 (7.1)	0 (0)	0 (0)	
Rain water tank onsite	1 (1.5)	1 (3.6)	0 (0)	0 (0)	
Other	1 (1.5)	1 (3.6)	0 (0)	0 (0)	
*Main handwashing facility for children*					
None	1 (1.5)	1 (3.6)	0 (0)	0 (0)	0.191
Tap	39 (57.4)	12 (42.9)	14 (60.9)	13 (76.5)	
Tippytap	13 (19.1)	5 (17.9)	6 (26.1)	2 (11.8)	
Bowl/bucket	15 (22.1)	10 (35.71)	3 (13.0)	2 (11.8)	
*% ELP with outdoor play area*	59 (86.8)	24 (85.7)	20 (86.9)	15 (88.2)	0.912
*Dust samples (% detected)*					
Pb	100 (136/136)	100 (55/55)	100 (47/47)	100 (34/34)	
As	93.4 (127/136)	94.5 (52/55)	91.5 (43/47)	94.1 (32/34)	
Cd	8.8 (12/136)	7.3 (4/55)	12.8 (6/47)	5.9 (2/34)	
*Soil samples (mg/kg)*					
Lead (Pb)					
% > LOD	91.8				
Mean ± SD	26.4 ± 27.3	24.9 ± 23.2	18.9 ± 20.3	43.8 ± 38.7	0.006
Mean (95% CI)	26.4 (21.5–31.3)	24.9 (18.5–31.5)	18.9 (12.8–25.0)	43.8 (27.5–60.1)	
Median [IQR]	16.0 [2.5–101]	16.0 [7.0–60.0]	13.0 [2.5–41.0]	32.0 [9.0–93.0]	
Range	2.5–159	2.5–121	2.5–122	2.5–159	
% > South African Pb soil reference values (230 mg/kg)	0	0	0	0	
% > Canadian reference soil values (61 mg/kg) (CCME, 2025)	9.0 (11/122)	5.7 (3/53)	2.2 (1/45)	29.2 (7/24)	
% > sample Pb mean	31.1 (38/122)	30.2 (16/53)	20.0 (9/45)	54.2 (13/24)	
IEUBK predicted blood Pb geometric mean levels (95% CI)	1.72 (0.69–4.31)				
% > CDC cut-off of 3.5 µg/dL	6.6				
Arsenic (As)					
% > LOD	95.1				
Mean ± SD	7.7 ± 9.4	9.8 ± 11.1	6.2 ± 8.8	5.8 ± 5.1	0.002
Mean (95% CI)	7.7 (6.0–9.4)	9.8 (6.8–12.9)	6.2 (3.6–8.8)	5.8 (3.7–7.9)	
Median [IQR]	4.4 [0.25–36.8]	6.2 [1.7–23.6]	2.7 [0.25–19.9]	4.6 [1.6–9.3]	
Range	0.25–61.4	0.6–61.4	0.25–42.4	0.5–25.3	
Hazard quotient (HQ) for As	0.023				
As lifetime excess cancer risk (2 years exposure duration)^**^	2.21 × 10^–4^				
As lifetime excess cancer risk (3 years exposure duration)^**^	3.32 × 10^–4^				
% > South African As soil reference values (48 mg/kg)	0.8 (1/122)	1.9 (1/53)	0	0	
% > Canadian reference soil values (18 mg/kg)	10.7 (13/122)	13.2 (7/53)	11.1 (5/45)	4.2 (1/24)	
% > sample As mean	31.1 (38/122)	39.6 (21/53)	24.4 (11/45)	25.0 (6/24)	
% Cd detected	16.4	11.3	15.6	29.2	0.114
* LOD – level of detection, SD – standard deviation ** Risk interpretation: EPA Acceptable Risk Range 1 × 10⁻⁶ to 1 × 10⁻^4^; above EPA benchmark					

**Fig. 2:**
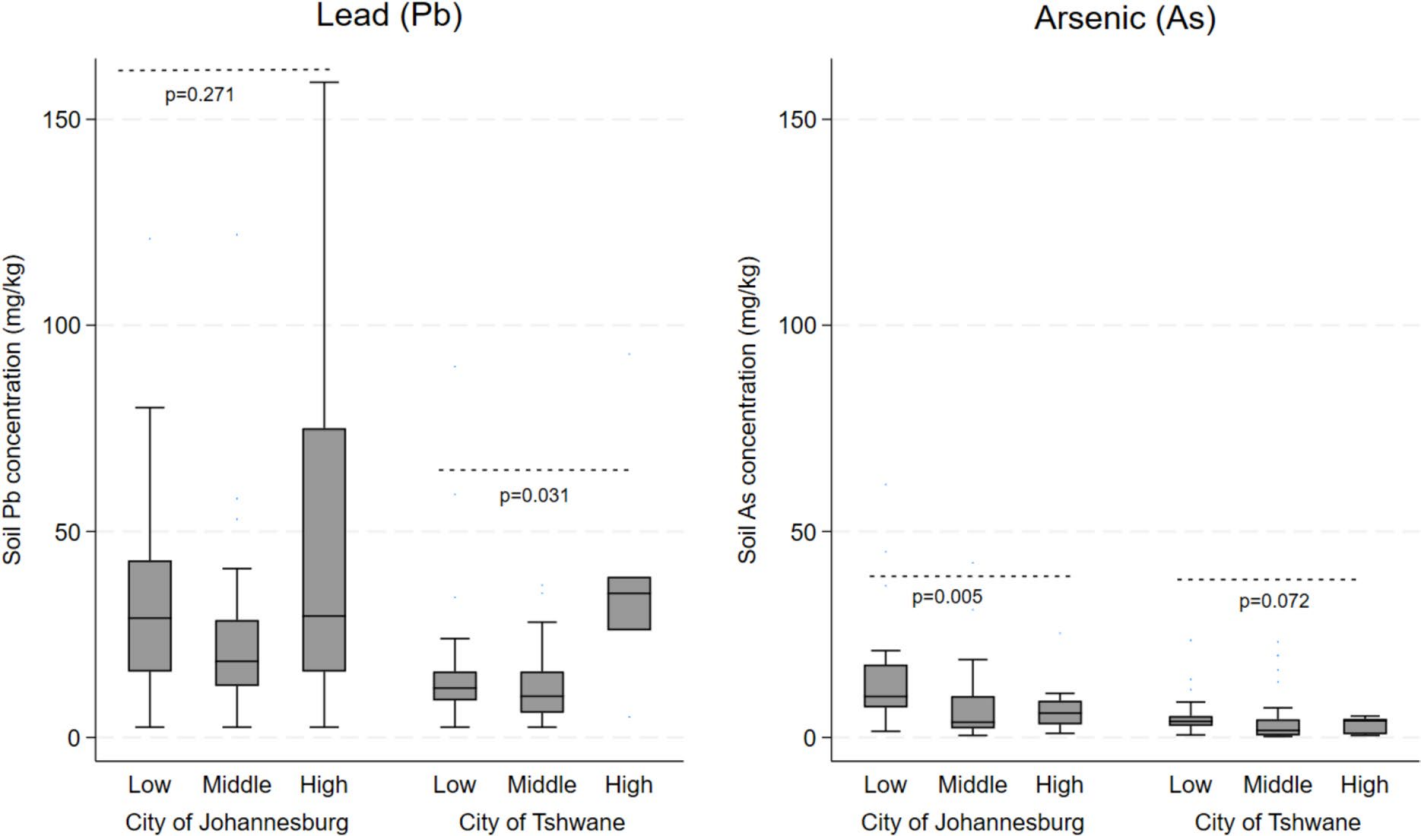
Boxplot graph of soil arsenic (As) and lead (Pb) levels (mg/kg) stratified by school fee band (low, middle and high) across two municipalities in Gauteng Province, South Africa

Mean concentrations of Pb in soil ranged from 24.9 mg/kg (95% CI, 18.5–31.5) in low-fee schools to 43.8 mg/kg (95% CI, 27.5–60.1) in high-fee schools (p = 0.006) (Table 1, Fig. 2). All soil samples were below the South African reference levels for lead (230 mg/kg) while 9% of samples were above the revised 2025 Canadian soil reference values (61 mg/kg) (CCME, 2025). Dust Pb was detected in 100% of samples across all sites and fee bands.

For As, 95.1% of soil samples were above the limit of detection (LOD). Mean soil As concentrations differed across fee bands (p = 0.002), with low-fee schools showing highest levels (9.8 mg/kg, 95% CI 6.8–12.9) compared to high fee band (5.8 mg/kg, 95% CI 3.7–7.9). Less than 1% of soil samples exceeded the South African soil guideline for As (48 mg/kg), (National Environmental Management: Waste Act, 2014, May 2) while 10.7% (13/122) exceeded the Canadian guideline (18 mg/kg). Above-threshold values were most frequent in low-fee schools (13.2%), followed by middle-fee (11.1%) and high-fee schools (4.2%). Cd was detected in a small proportion of soil and dust samples across all municipalities and fee bands, indicating generally low prevalence.

The estimated average daily intake (ADI) of As was below the corresponding reference dose (RfD), resulting in hazard quotients (HQ) of 0.023 for As, indicating minimal non-carcinogenic risk via this exposure pathway. Hazard quotients for Cd were not calculated because approximately 84% of Cd concentrations were below limit of detection limiting reliable quantitative risk estimation. For children exposed to As at school for approximately 2 years, the estimated combined lifetime cancer risk was 2.2 × 10⁻^4^, exceeding EPA’s acceptable risk threshold of 1 × 10⁻^4^. Bladder and lung cancers were considered, with lung cancer (1.49 × 10⁻^4^) contributing slightly more to the combined risk. Although exposure was limited to a short period of childhood, linear dose–response assumptions indicate that even partial lifetime exposure results in a measurable excess cancer risk, highlighting the need for continued monitoring and mitigation of As in school soils.

The IEUBK model predicted a population geometric mean blood Pb level of 1.72 µg/dL (95% CI 0.69–4.31) for children aged 4–5 years attending preschool in Gauteng, with 6.6% of predicted blood levels exceeding 3.5 µg/dL CDC threshold. Predicted geometric mean blood Pb levels were slightly higher for City of Johannesburg (1.76 µg/dL, 95% CI 0.70–4.42) than Tshwane (1.68 µg/dL, 95% CI 0.67–4.22), with 7.2% and 5.9% of blood levels exceeding the 3.5 µg/dL CDC threshold. These findings suggest that children’s exposure to Pb in soil could potentially be associated with low-level blood Pb levels in children, with some exceeding the CDC threshold.

**Fig. 3:**
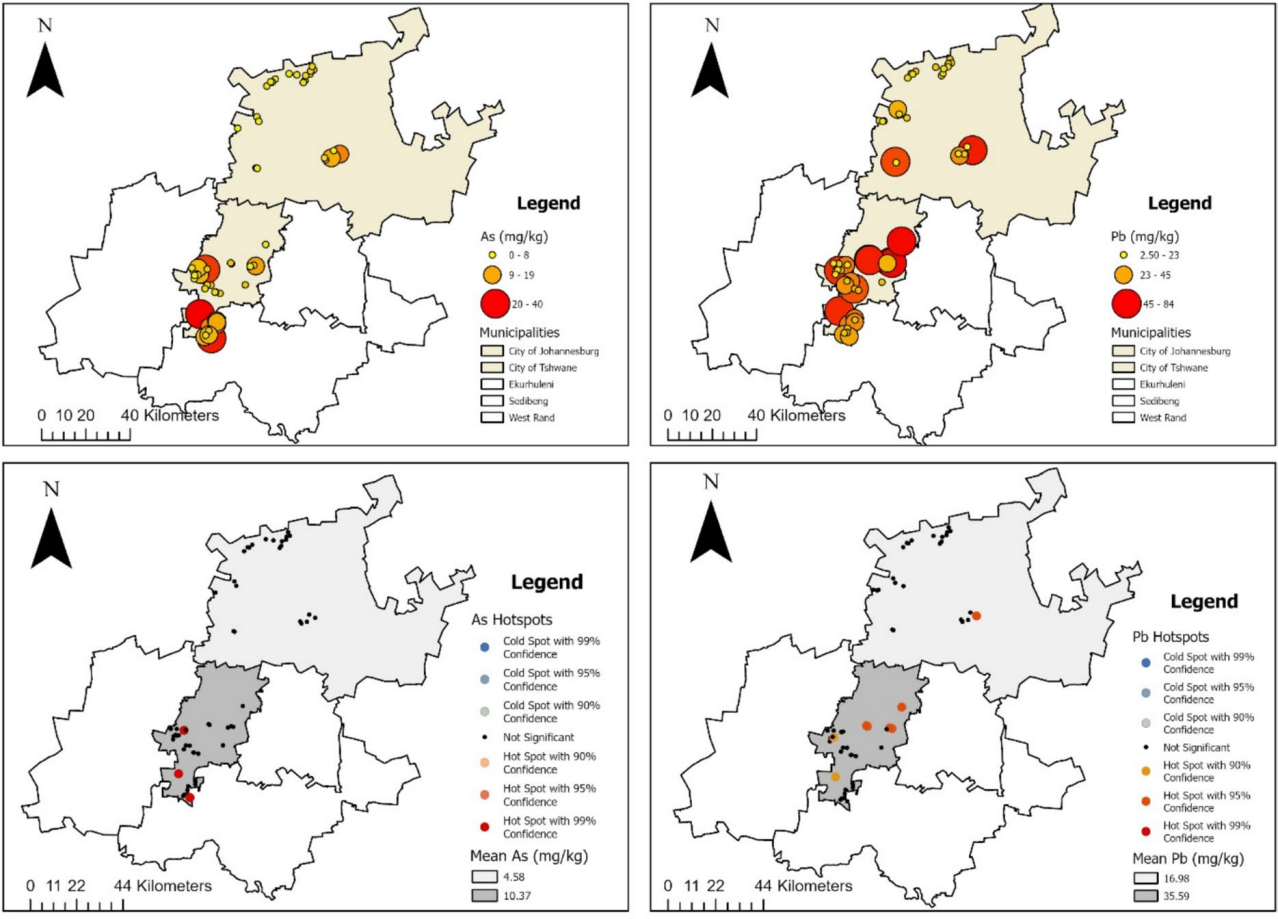
[Top] The spatial distribution of As and Pb in ELP playground soils across two municipalities in Gauteng. Each circle represents the mean heavy metal concentration in each site, with larger-sized dots indicating greater heavy metal content in soils. [Bottom] Hotspot analysis of As and Pb across study area, showing areas with elevated levels relative to background

Figure 3 (top) shows the spatial distribution of As and Pb in ELP playground soils across two municipalities, with larger sized dots indicating greater heavy metal content in soils. Higher levels of As and Pb were observed in City of Johannesburg compared to City of Tshwane municipality. Figure 3 (bottom) shows elevated concentrations of As clustered primarily in the Johannesburg region, while Pb hotspots extend from Johannesburg into Tshwane. Hotspot analysis also confirmed these patterns, significant As hotspots (90–99% confidence levels) were observed in localised areas of Johannesburg, while most other sampling sites were not significant or fell within cold spot zones. In contrast, Pb hotspots were more spatially extensive, with significant clusters identified in both Johannesburg and Tshwane municipalities. Cold spots for Pb were detected in peripheral areas, indicating lower concentrations relative to the mean. Overall, Johannesburg consistently emerged as a contamination hotspot. Pb displayed broader spatial spread and more pronounced hotspots compared to As, suggesting a greater potential for widespread exposure.

**Fig. 4:**
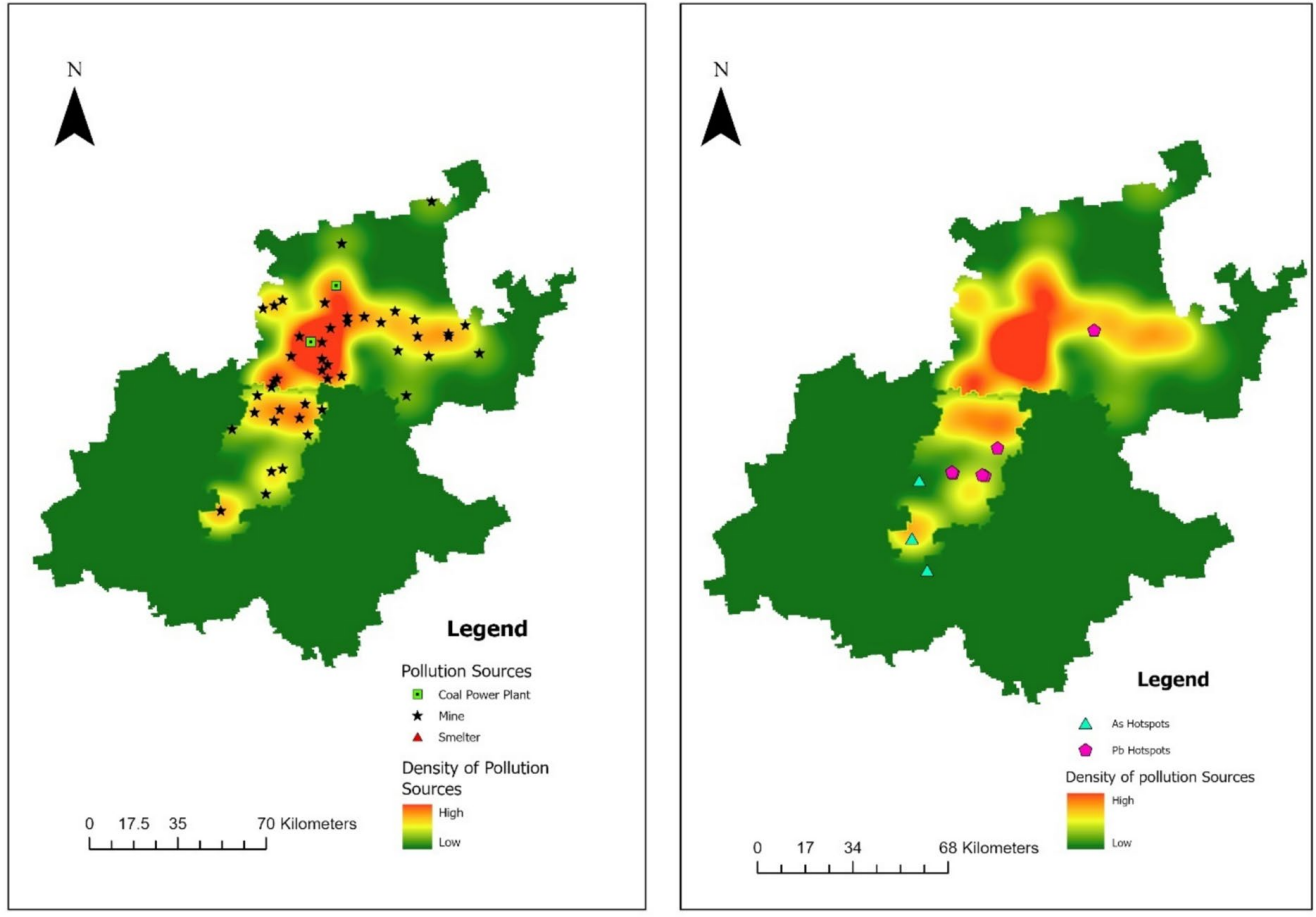
Spatial density of pollution sources (left) and overlap with arsenic (As) and lead (Pb) hotspots (95 – 99% confidence) (right) across the study area

Figure 4 shows the spatial relationship between major pollution sources and metal hotspots. The density map of pollution sources (left) indicates a high concentration of mines in the central and southern parts of the study area, particularly around Johannesburg and its surroundings. This clustering coincides with the distribution of As and Pb hotspots (right), though causation cannot be inferred. Pb hotspots were more spatially widespread compared to As hotspots, and the observed patterns may reflect multiple contributing factors, including industrial and mining activities, legacy building materials, and other unmeasured sources such as traffic.

## Discussion

This study assessed environmental metal exposure risks in ELPs across two municipalities in Gauteng Province, South Africa, using dust and soil samples from indoor and outdoor areas, while also examining potential sources of pollution. Hotspot analysis showed that elevated As and Pb concentrations in the City of Johannesburg and Tshwane municipalities tended to coincide with areas of higher density of potential pollution sources, including proximity to mining activities. Interestingly, elevated Pb levels were observed in some high fee band schools, whereas higher As concentrations were more widespread distribution and observed in lower fee paying ELPs. Across sites, the non-carcinogenic hazard quotients calculated for As (HQ = 0.023) and Cd (interpreted qualitatively due to high censoring) indicate low health risk through soil ingestion, while the lifetime cancer excess risks from As exposure suggest potential long-term carcinogenic concern. In addition, the IEUBK model predicted a geometric mean blood Pb level of 1.72 µg/dL (95% CI 0.69–4.31), with 6.6% exceeding the CDC blood lead reference value of 3.5 µg/dL, among children aged 4–5 year. These findings suggest that children’s exposure to Pb contaminated soil at schools could potentially be associated with low level blood Pb levels. Spatial distribution of soil metal concentrations and associated health risks suggest that legacy industrial and mining-related activities may influence contamination patterns, highlighting the importance of monitoring ELPs across all socioeconomic levels.

Our study found localised hotspots of elevated levels of As and Pb across ELPs in the City of Johannesburg and Tshwane, with patterns differing by socioeconomic groups defined through school fee bands. Comparable studies in South Africa have reported that while average exposure levels in schools and childcare settings often fall below health-based thresholds, localised contamination remains a concern, particularly in areas near mining and industrial activities (Kootbodien et al., 2012; Mathee et al., 2018, 2022; Shezi et al., 2022).

Several studies in Gauteng have reported elevated As and Pb in soils surrounding mine tailings, underscoring the role of legacy mining in driving localised community contamination. In South Africa, Mathee and colleagues (2018) reported Pb and As levels in garden soils exceeding South African and Canadian guidelines in Johannesburg’s inner city and near mine tailings facilities (Mathee et al., 2018), and later showed that both soil and blood lead levels were higher among participants living closer to mine tailings compared to those living further away (Mathee et al., 2022). Similarly, elevated soil As concentration were reported from a Johannesburg school garden located near mine tailings (Kootbodien et al., 2012). Additional studies have also reported elevated As and Pb in soil around mine tailings in Gauteng Province (Okereafor et al., 2019), highlighting the influence of legacy mining activities on local contamination in nearby communities. In addition, elevated Pb in soil and other trace metal levels near areas of dense traffic and industrial activities were reported in Tshwane (Olowoyo et al., 2010). While our study did not specifically assess traffic-related pollution, this remains an important potential contributor that should be considered in future assessments. Differences in soil metal levels across fee bands may be influenced by multiple sources: higher As in low-fee schools could reflect legacy industrial and mining contamination, whereas elevated lead in high-fee schools may originate from building materials such as old paint or plumbing. While this study did not directly attribute sources, these patterns show that multiple exposure pathways can drive metal contamination in children’s environments. Further investigation is needed to confirm these source attributions in the study population. Taken together, these findings are consistent with our hotspot analysis, which suggests that legacy mining and other localised environmental sources continue to shape spatial patterns of soil contamination in urban areas.

### Strength and limitations

Although the sample size was relatively small, this study included the use of GIS-based hotspot and kernel density estimation to identify potential spatial patterns of elevated metal levels, which improved the detection of localised exposure risks. In addition, school fee bands provided a useful proxy for socioeconomic status, allowing comparison of metal exposures across different community settings. A limitation of the study is that dust measurements were not collected using standardised area-based wipe methods, which may have underestimated children’s exposure; future studies could be strengthened by adopting this approach to allow direct comparison with EPA dust-lead guidelines. In addition, although the study used established reference values for risk calculations, it did not include biomonitoring (e.g., blood lead levels), or hand wipes which limit the ability to assess internal dose or validate environmental exposure estimates.

## Conclusion

This exploratory environmental assessment found that non-carcinogenic risk to children from soil As exposure was generally low. However, lifetime cancer risk from As exceeded the US EPA benchmark in some schools, and Pb risk, evaluated using the IEUBK model, indicated potential exposure concerns. Hotspot analysis identified localised areas of elevated As and Pb levels that varied across ELP socioeconomic levels, highlighting the importance of ongoing monitoring to ensure safe learning environments for children across all school socioeconomic levels.

## Competing interests

The authors declare no competing interests.

## Ethics approval

Permission to conduct the national Thrive by Five Index 2024 study was obtained from the National Department of Basic Education. Ethical approval was obtained from the Ethics Review Committee of the Faculty of Humanities, University of Cape Town (PSY2024-032), and the Human Research Ethics Committee (HREC) of the South African Medical Research Council (SAMRC).

## Supplementary Information

Below is the link to the electronic supplementary material.

Supplementary file1 (DOCX 28 kb)

## Data Availability

Data will be made available upon reasonable request through DataDrive2030.
