# Supplementary material for: Assessment of soil metal exposure, associated health risks and indoor dust screening in early learning programmes in Gauteng Province, South Africa
Source: Environ Geochem Health. 2026 Feb 4;48(3):136. doi: 10.1007/s10653-026-03034-w (PMC12872739; doi:10.1007/s10653-026-03034-w)
Supplement: Supplementary file 1 — Supplementary file1 (DOCX 28 kb) [file 10653_2026_3034_MOESM1_ESM.docx]

Table S1. Elemental concentration of standard reference material SRM 2583 (n=3, mean ± SD)

| **Element** | **Measured value (mg kg^-1^)** | **Certified value (mg kg^-1)^)** | **Recovery (%)** |
| --- | --- | --- | --- |
| Cd | 6.46 ± 0.67 | 7.3 ± 3.7 | 88 |
| Cr | 54.97 ± 3.14 | 80 ± 22 | 69 |
| Pb | 71.69 ± 6.57 | 85.9 ± 7.2 | 84 |

Table S2. Metal concentrations in soil samples

| ID | Arsenic | Cadmium | Lead |
| --- | --- | --- | --- |
| Units | mg/kg | mg/kg | mg/kg |
| Detection Limit | <0.5 | <0.1 | <5 |
| 101-SOIL1 | 2.1 | <0.1 | 8 |
| 101-SOIL2 | 2.7 | <0.1 | 8 |
| 102-SOIL1 | 10.3 | <0.1 | 10 |
| 102-SOIL2 | 11.3 | <0.1 | 14 |
| 103-SOIL1 | 1.1 | 0.3 | 14 |
| 103-SOIL2 | 0.5 | 0.2 | 12 |
| 104-SOIL1 | 2.2 | <0.1 | 7 |
| 104-SOIL2 | 2.5 | <0.1 | <5 |
| 105-SOIL1 | 3.4 | 0.4 | 16 |
| 105-SOIL2 | 7.7 | 0.2 | 121 |
| 107-SOIL1 | 8.4 | <0.1 | 15 |
| 107-SOIL2 | 8.8 | <0.1 | 8 |
| 108-SOIL1 | 61.4 | <0.1 | 45 |
| 108-SOIL2 | 1.5 | <0.1 | <5 |
| 109-SOIL1 | 9.3 | <0.1 | 18 |
| 109-SOIL2 | 9.8 | <0.1 | 13 |
| 110-SOIL1 | 8.6 | <0.1 | 11 |
| 110-SOIL2 | 8.1 | <0.1 | 7 |
| 112-SOIL1 | 6.8 | <0.1 | 33 |
| 112-SOIL2 | 3.5 | <0.1 | 35 |
| 113-SOIL1 | 7.7 | <0.1 | 34 |
| 113-SOIL2 | 6.7 | <0.1 | 48 |
| 114-SOIL1 | 1 | <0.1 | 9 |
| 114-SOIL2 | 10.3 | <0.1 | 23 |
| 115-SOIL1 | 3.5 | 0.1 | 16 |
| 115-SOIL2 | 7.3 | <0.1 | 26 |
| 116-SOIL1 | 4.8 | <0.1 | 24 |
| 116-SOIL2 | 4.2 | 0.2 | 22 |
| 117-SOIL1 | 2.8 | 0.1 | 53 |
| 117-SOIL2 | 4.3 | <0.1 | 58 |
| 118-SOIL1 | 4.7 | <0.1 | 19 |
| 118-SOIL2 | 10.7 | <0.1 | 19 |
| 119-SOIL1 | 3.2 | <0.1 | 93 |
| 119-SOIL2 | 25.3 | <0.1 | 70 |
| 120-SOIL1 | 1.6 | <0.1 | 9 |
| 120-SOIL2 | 8.9 | 0.2 | 159 |
| 122-SOIL1 | 0.8 | <0.1 | <5 |
| 122-SOIL2 | 3.2 | <0.1 | 24 |
| 123-SOIL1 | 5.4 | <0.1 | 47 |
| 123-SOIL2 | 9.3 | 0.1 | 101 |
| 124-SOIL1 | 7.9 | 0.2 | 75 |
| 124-SOIL2 | 6.4 | 0.3 | 75 |
| 125-SOIL1 | 13 | <0.1 | 23 |
| 125-SOIL2 | 12.7 | <0.1 | 16 |
| 126-SOIL1 | 13.6 | <0.1 | 37 |
| 126-SOIL2 | 15 | <0.1 | 36 |
| 127-SOIL1 | 18.2 | <0.1 | 43 |
| 127-SOIL2 | 18.2 | <0.1 | 40 |
| 128-SOIL1 | 11.1 | <0.1 | 26 |
| 128-SOIL2 | 11.4 | <0.1 | 32 |
| 129-SOIL1 | 5.5 | <0.1 | 19 |
| 129-SOIL2 | 7.3 | <0.1 | 21 |
| 130-SOIL1 | 3.4 | <0.1 | 13 |
| 130-SOIL2 | 4.5 | <0.1 | 19 |
| 131-SOIL1 | 2.3 | <0.1 | 14 |
| 131-SOIL2 | 18.9 | <0.1 | 19 |
| 132-SOIL1 | 42.4 | <0.1 | 31 |
| 132-SOIL2 | 31 | <0.1 | 26 |
| 133-SOIL1 | 17.7 | <0.1 | 45 |
| 133-SOIL2 | 21.1 | <0.1 | 40 |
| 134-SOIL1 | 36.8 | <0.1 | 80 |
| 134-SOIL2 | 45.1 | <0.1 | 60 |
| 135-SOIL1 | 2.1 | 0.8 | 41 |
| 135-SOIL2 | 2.5 | 0.4 | 122 |
| 136-SOIL1 | 4.2 | <0.1 | 13 |
| 136-SOIL2 | 2.8 | <0.1 | 12 |
| 137-SOIL1 | 4.7 | <0.1 | 11 |
| 138-SOIL1 | 7.2 | <0.1 | 13 |
| 138-SOIL2 | 3.9 | <0.1 | 10 |
| 139-SOIL1 | 5.1 | <0.1 | 15 |
| 139-SOIL2 | 5 | <0.1 | 19 |
| 140-SOIL1 | 4.7 | 1.5 | 9 |
| 140-SOIL2 | 11.6 | <0.1 | 18 |
| 141-SOIL1 | 3.6 | <0.1 | 12 |
| 141-SOIL2 | 3.2 | <0.1 | 12 |
| 142-SOIL1 | 6.4 | <0.1 | 11 |
| 142-SOIL2 | 5.2 | <0.1 | 13 |
| 143-SOIL1 | 3.5 | <0.1 | 8 |
| 143-SOIL2 | 6.2 | <0.1 | 12 |
| 144-SOIL1 | 1.7 | <0.1 | <5 |
| 144-SOIL2 | 2.7 | <0.1 | 6 |
| 145-SOIL1 | 1.7 | <0.1 | 8 |
| 145-SOIL2 | 4.1 | <0.1 | 28 |
| 146-SOIL1 | 3 | <0.1 | 10 |
| 146-SOIL2 | 2.6 | 0.2 | 24 |
| 147-SOIL1 | 2.9 | <0.1 | 9 |
| 147-SOIL2 | 2 | 0.2 | 10 |
| 148-SOIL1 | 1.7 | <0.1 | 13 |
| 148-SOIL2 | 2.2 | <0.1 | 17 |
| 149-SOIL1 | 0.9 | <0.1 | 16 |
| 149-SOIL2 | 1.4 | <0.1 | 8 |
| 150-SOIL1 | <0.5 | <0.1 | 6 |
| 150-SOIL2 | <0.5 | 0.3 | 10 |
| 151-SOIL1 | <0.5 | <0.1 | 9 |
| 151-SOIL2 | <0.5 | <0.1 | <5 |
| 152-SOIL1 | 8.6 | <0.1 | 12 |
| 152-SOIL2 | 3.5 | <0.1 | 7 |
| 153-SOIL1 | 4.7 | 0.2 | 20 |
| 153-SOIL2 | 4.7 | <0.1 | 19 |
| 155-SOIL1 | 5.2 | <0.1 | 32 |
| 155-SOIL2 | 4.5 | <0.1 | 39 |
| 156-SOIL1 | 0.7 | <0.1 | <5 |
| 156-SOIL2 | 13.5 | <0.1 | 35 |
| 157-SOIL1 | 19.9 | <0.1 | 20 |
| 157-SOIL2 | 4.4 | <0.1 | 10 |
| 158-SOIL1 | 4.4 | 0.4 | 38 |
| 159-SOIL1 | 14.1 | <0.1 | 59 |
| 159-SOIL2 | 23.6 | <0.1 | 90 |
| 161-SOIL1 | 0.5 | <0.1 | 5 |
| 163-SOIL1 | 23.2 | <0.1 | 28 |
| 163-SOIL2 | 16.4 | <0.1 | 37 |
| 164-SOIL1 | 0.5 | <0.1 | <5 |
| 165-SOIL1 | 3.7 | <0.1 | 26 |
| 165-SOIL2 | 0.8 | 0.3 | 93 |
| 166-SOIL1 | 0.6 | <0.1 | 7 |
| 166-SOIL2 | 0.8 | <0.1 | 14 |
| 167-SOIL1 | 4.4 | <0.1 | 16 |
| 167-SOIL2 | 4.2 | 0.4 | 34 |
| 168-SOIL1 | <0.5 | <0.1 | 12 |
| 168-SOIL2 | <0.5 | <0.1 | <5 |
| 169-SOIL1 | 3.2 | <0.1 | 8 |
| 169-SOIL2 | 3.1 | <0.1 | 14 |
| 171-SOIL1 | 1.2 | <0.1 | <5 |
| 171-SOIL2 | 1.9 | <0.1 | <5 |

Table S2. Metal concentrations in dust samples

| Dust sample ID | As (Arsenic) | Cd (Cadmium) | Pb (Lead) |
| --- | --- | --- | --- |
| Units | mg/kg | mg/kg | mg/kg |
| Detection Limit | <0.001 | <0.001 | <0.001 |
| 101_SD1 | <0.001 | <0.001 | 0.995 |
| 101_SD2 | 0.620 | 0.138 | 28 |
| 102_SD1 | 0.905 | 0.036 | 2.34 |
| 102_SD2 | 3.238 | <0.001 | 14 |
| 103_SD1 | 0.092 | 0.099 | 28 |
| 103_SD2 | 3.08 | 0.083 | 39 |
| 104_SD1 | 0.065 | <0.001 | 7.31 |
| 104_SD2 | <0.001 | <0.001 | 3.42 |
| 105_SD1 | <0.001 | 0.026 | 5.98 |
| 105_SD2 | <0.001 | 0.075 | 1.97 |
| 106_SD1 | <0.001 | 0.012 | 4.36 |
| 106_SD2 | 0.977 | 0.024 | 6.69 |
| 107_SD1 | <0.001 | -0.009 | 4.25 |
| 107_SD2 | 3.78 | 0.536 | 17 |
| 108_SD1 | 7.01 | <0.001 | 4.78 |
| 108_SD2 | 6.92 | <0.001 | 5.38 |
| 109_SD1 | <0.001 | <0.001 | 0.328 |
| 109_SD2 | 0.478 | <0.001 | 6.97 |
| 110_SD1 | 0.981 | <0.001 | 11 |
| 110_SD2 | 0.377 | <0.001 | 48 |
| 111_SD1 | 0.363 | <0.001 | 11 |
| 111_SD2 | 0.535 | <0.001 | 9.07 |
| 112_SD1 | 0.763 | <0.001 | 10 |
| 112_SD2 | <0.001 | <0.001 | 8.59 |
| 113_SD1 | 1.52 | <0.001 | 68 |
| 113_SD2 | 1.32 | <0.001 | 11 |
| 114_SD1 | 0.719 | <0.001 | 9.21 |
| 114_SD2 | 0.639 | <0.001 | 26 |
| 115_SD1 | 0.202 | <0.001 | 2.87 |
| 115_SD2 | 0.509 | <0.001 | 10 |
| 116_SD1 | 0.151 | <0.001 | 1.59 |
| 116_SD2 | 0.277 | <0.001 | 1.99 |
| 117_SD1 | 1.03 | <0.001 | 21 |
| 118_SD1 | 0.696 | <0.001 | 17 |
| 118_SD2 | 0.869 | <0.001 | 34 |
| 119_SD1 | 0.108 | <0.001 | 4.99 |
| 119_SD2 | 0.120 | <0.001 | 4.43 |
| 120_SD1 | 0.931 | <0.001 | 26 |
| 120_SD2 | 1.27 | <0.001 | 51 |
| 121_SD1 | 0.271 | <0.001 | 5.05 |
| 121_SD2 | 0.027 | <0.001 | 3.94 |
| 122_SD1 | 0.150 | <0.001 | 12 |
| 122_SD2 | 0.212 | <0.001 | 9.85 |
| 123_SD1 | 0.292 | <0.001 | 6.30 |
| 123_SD2 | 0.099 | <0.001 | 3.30 |
| 124_SD1 | 0.180 | <0.001 | 5.87 |
| 124_SD2 | 0.394 | <0.001 | 6.41 |
| 125_SD1 | 0.267 | <0.001 | 1.47 |
| 125_SD2 | 0.107 | <0.001 | 0.888 |
| 126_SD1 | 0.263 | <0.001 | 1.61 |
| 127_SD1 | 0.392 | <0.001 | 2.51 |
| 127_SD2 | 0.390 | <0.001 | 1.74 |
| 128_SD1 | 0.618 | <0.001 | 3.20 |
| 128_SD2 | 0.483 | <0.001 | 3.31 |
| 129_SD1 | 0.743 | <0.001 | 2.37 |
| 129_SD2 | 1.844 | <0.001 | 6.06 |
| 130_SD1 | 1.37 | <0.001 | 2.63 |
| 130_SD2 | 0.143 | <0.001 | 0.382 |
| 131_SD1 | 0.062 | <0.001 | 2.96 |
| 131_SD2 | 0.539 | <0.001 | 3.73 |
| 132_SD1 | 0.459 | <0.001 | 6.50 |
| 132_SD2 | 0.301 | <0.001 | 0.934 |
| 133_SD1 | 1.40 | <0.001 | 3.86 |
| 133_SD2 | 1.39 | <0.001 | 4.02 |
| 134_SD1 | 1.25 | <0.001 | 3.08 |
| 134_SD2 | 1.51 | <0.001 | 16 |
| 135_SD1 | 0.716 | <0.001 | 39 |
| 135_SD2 | 0.314 | <0.001 | 18 |
| 136_SD1 | 1.45 | <0.001 | 25 |
| 136_SD2 | 0.622 | <0.001 | 7.04 |
| 137_SD1 | 0.630 | <0.001 | 3.75 |
| 137_SD2 | 1.25 | 0.336 | 8.51 |
| 138_SD1 | 1.62 | <0.001 | 14 |
| 138_SD2 | 0.808 | <0.001 | 87 |
| 139_SD1 | 1.03 | <0.001 | 9.80 |
| 139_SD2 | 0.325 | <0.001 | 2.84 |
| 140_SD1 | 0.748 | <0.001 | 23 |
| 140_SD2 | 1.11 | <0.001 | 4.54 |
| 141_SD1 | 0.923 | <0.001 | 5.27 |
| 141_SD2 | 0.265 | <0.001 | 23 |
| 142_SD1 | 0.363 | <0.001 | 1.85 |
| 142_SD2 | 0.183 | <0.001 | 1.48 |
| 143_SD1 | 0.467 | <0.001 | 2.01 |
| 143_SD2 | 0.555 | <0.001 | 2.30 |
| 144_SD1 | 0.221 | 0.289 | 9.43 |
| 144_SD2 | 0.396 | <0.001 | 3.20 |
| 145_SD1 | 0.319 | <0.001 | 4.73 |
| 145_SD2 | 0.501 | <0.001 | 5.71 |
| 146_SD1 | 0.690 | <0.001 | 9.76 |
| 146_SD2 | 0.684 | <0.001 | 5.21 |
| 147_SD1 | 0.563 | <0.001 | 3.50 |
| 147_SD2 | 0.416 | <0.001 | 1.92 |
| 148_SD1 | 0.337 | <0.001 | 8.40 |
| 148_SD2 | 0.299 | <0.001 | 4.03 |
| 149_SD1 | 1.07 | <0.001 | 24 |
| 149_SD2 | 0.412 | <0.001 | 9.22 |
| 150_SD1 | 0.465 | <0.001 | 6.35 |
| 150_SD2 | <0.001 | <0.001 | 1.64 |
| 151_SD1 | 0.605 | <0.001 | 6.73 |
| 151_SD2 | 0.087 | <0.001 | 4.78 |
| 152_SD1 | 0.528 | <0.001 | 14 |
| 152_SD2 | 0.340 | <0.001 | 4.30 |
| 153_SD1 | 0.525 | <0.001 | 5.67 |
| 153_SD2 | 0.860 | <0.001 | 7.29 |
| 154_SD1 | 0.140 | <0.001 | 196 |
| 154_SD2 | 0.235 | <0.001 | 45 |
| 155_SD1 | 0.147 | <0.001 | 4.84 |
| 155_SD2 | 0.110 | <0.001 | 2.48 |
| 156_SD1 | 0.166 | <0.001 | 0.882 |
| 156_SD2 | 0.130 | <0.001 | 0.597 |
| 157_SD1 | 0.182 | <0.001 | 1.75 |
| 157_SD2 | 0.201 | <0.001 | 2.60 |
| 158_SD1 | 0.566 | <0.001 | 6.57 |
| 158_SD2 | 0.074 | <0.001 | 3.49 |
| 159_SD1 | 0.335 | <0.001 | 2.51 |
| 159_SD2 | 0.163 | <0.001 | 1.40 |
| 160_SD1 | 2.70 | 0.016 | 25 |
| 160_SD2 | 2.93 | 0.034 | 24 |
| 161_SD1 | 0.146 | <0.001 | 1.66 |
| 161_SD2 | 0.147 | <0.001 | 2.69 |
| 162_SD1 | 0.176 | <0.001 | 7.40 |
| 162_SD2 | 0.058 | <0.001 | 2.33 |
| 163_SD1 | 2.41 | <0.001 | 6.80 |
| 163_SD2 | 0.200 | <0.001 | 2.20 |
| 164_SD1 | 0.163 | <0.001 | 2.24 |
| 164_SD2 | 0.056 | <0.001 | 0.498 |
| 165_SD1 | 0.469 | <0.001 | 2.58 |
| 165_SD2 | 0.249 | <0.001 | 1.40 |
| 166_SD1 | 0.194 | <0.001 | 3.28 |
| 166_SD2 | 0.368 | <0.001 | 2.65 |
| 167_SD1 | 0.898 | <0.001 | 8.57 |
| 167_SD2 | 0.312 | <0.001 | 3.54 |
| 168_SD1 | 0.168 | <0.001 | 2.30 |
| 168_SD2 | 0.076 | <0.001 | 0.295 |
| 169_SD1 | 0.486 | <0.001 | 2.71 |
| 169_SD2 | 0.129 | <0.001 | 3.46 |
| 170_SD1 | 0.198 | <0.001 | 3.01 |
| 170_SD2 | 0.248 | <0.001 | 6.70 |
| 171_SD1 | 0.181 | <0.001 | 6.75 |
| 171_SD2 | 0.371 | <0.001 | 2.75 |
| SD = Surface dust | | | |
